# Editorial: Molecular Regulation and Therapeutic Potential of Thermogenic Fat Cells

**DOI:** 10.3389/fendo.2016.00026

**Published:** 2016-03-29

**Authors:** Jun Wu

**Affiliations:** ^1^Life Sciences Institute, University of Michigan Medical School, Ann Arbor, MI, USA; ^2^Department of Molecular and Integrative Physiology, University of Michigan Medical School, Ann Arbor, MI, USA

**Keywords:** brown fat, beige fat, obesity, adaptive thermogenesis, metabolic diseases

**The Editorial on the Research Topic**

**Molecular Regulation and Therapeutic Potential of Thermogenic Fat Cells**

Much of systemic metabolism depends on the activity and function of adipose tissue. Adipocytes in mammals fall into two categories classified by their primary functions: white fat cells that mediate energy storage and thermogenic fat cells that counteract hypothermia and obesity through adaptive thermogenesis. Whereas white fat and its function as an energy reservoir and endocrine organ have been studied for decades and are relatively well understood, until recently, many aspects of thermogenic fat biology have remained elusive.

It has long been appreciated that the thermogenic brown adipose tissue that exists in the interscapular depots of small rodents, hibernating mammals, and human infants provides significant evolutionary advantages against cold stress (1). The last decade has witnessed an explosion of interest and studies focusing on the regulation of thermogenic fat and potential therapeutics targeting these adipocytes. Some groundbreaking discoveries have led to our current understanding of thermogenic fat biology. Accumulating evidence supports the hypothesis that thermogenic fat cells arise from at least two different developmental origins: the ones of a skeletal muscle-like lineage are now called “classical” brown fat cells, and a new type of thermogenic fat cell that is interspersed within white adipose tissue, which is normally referred to as the beige/brite (brown in white) fat cell. Definitions of types and/or subtypes of thermogenic fat cells will continue to evolve as further studies reveal distinctive functions of these cells. The rediscovery of thermogenic fat cells in human adults ignited enthusiasm toward these cells serving as novel therapeutic targets against obesity and its associated metabolic disorders. One of the key questions is whether the average amount and activity of these cells in the adult human is sufficient to influence whole body metabolism. Emerging data are starting to address this as several recent reports have demonstrated that activated thermogenic fat cells help to improve glucose tolerance and insulin sensitivity in humans.

In this research topic, leading experts in the energy metabolism field provide an overview of our current understanding of these metabolically active fat cells. We begin our discussions with mechanistic insights on the transcriptional regulation of thermogenic fat cells [Park et al.; Mueller; Bayindir et al.]. Reviews and a perspective article next explore how mitochondrial function, including redox homeostasis, is regulated within these thermogenic adipocytes [Nam and Cooper; Jeanson et al.; Ro et al.]. Central control of adaptive thermogenesis and other functions of thermogenic adipocytes are also highlighted [Yang and Ruan; Zhang and Bi]. Importantly, several articles illustrate that interconnections exist between adipose tissue function and overall metabolic health, and how to increase the content and activity of these cells to ultimately fulfill their therapeutic potential in humans [Lester et al.; Peng et al.; Porter et al.]. As technology has advanced, novel interdisciplinary approaches have been developed to harvest the thermogenic capacity in fat tissue toward the control of obesity and metabolic diseases [Jiang et al.; Tharp and Stahl]. We anticipate that all these articles will help the scientific community, both experts in the brown and beige fat area and newcomers entering the field to understand the molecular mechanisms of brown/beige fat development and induction, and appreciate the physiological impact of thermogenic fat activity. I would like to thank all the authors and reviewers for their contribution and discussion to put together this wonderful topic that may inspire further interest in this exciting new field.

## Author Contributions

JW conceived and wrote the manuscript.

## Conflict of Interest Statement

The author declares that the research was conducted in the absence of any commercial or financial relationships that could be construed as a potential conflict of interest.
